# Occupational exposure to particles and mitochondrial DNA - relevance for blood pressure

**DOI:** 10.1186/s12940-017-0234-4

**Published:** 2017-03-09

**Authors:** Yiyi Xu, Huiqi Li, Maria Hedmer, Mohammad Bakhtiar Hossain, Håkan Tinnerberg, Karin Broberg, Maria Albin

**Affiliations:** 1 Division of Occupational and Environmental Medicine, Laboratory Medicine, Lund University, 221 85 Lund, Sweden; 2 Unit of Metals & Health, Institute of Environmental Medicine, Karolinska Institutet, 171 77 Stockholm, Sweden; 3 Unit of Occupational Medicine, Institute of Environmental Medicine, Karolinska Institutet, 171 77 Stockholm, Sweden

**Keywords:** Particle, Mitochondria, Copy number, DNA methylation, Blood pressure

## Abstract

**Background:**

Particle exposure is a risk factor for cardiovascular diseases. Mitochondrial DNA (mtDNA) is a primary target for oxidative stress generated by particle exposure. We aimed to elucidate the effects of occupational exposure to particle-containing welding fumes on different biomarkers of mtDNA function, and in turn, explore if they modify the association between particle exposure and cardiovascular response, measured as blood pressure.

**Methods:**

We investigated 101 welders and 127 controls (all non-smoking males) from southern Sweden. Personal sampling of the welders’ exposure to respirable dust was performed during work hours (average sampling time: 6.8 h; range: 2.4-8.6 h) and blood pressure was measured once for each subject. We measured relative mtDNA copy number by quantitative PCR and methylation of the mitochondrial regulatory region D-loop and the tRNA encoding gene *MT-TF* by bisulfite-pyrosequencing. We calculated the relative number of unmethylated D-loop and *MT-TF* as markers of mtDNA function to explore the modification of mtDNA on the association between particle exposure and blood pressure. General linear models were used for statistical analyses.

**Results:**

Welders had higher mtDNA copy number (β = 0.11, *p* = 0.003) and lower DNA methylation of D-loop (β = −1.4, *p* = 0.002) and *MT-TF* (β = −1.5, *p* = 0.004) than controls. Higher mtDNA copy number was weakly associated with higher personal respirable dust exposure among welders with exposure level above 0.7 mg/m^3^ (β = 0.037, *p* = 0.054). MtDNA function modified the effect of welding fumes on blood pressure: welders with low mtDNA function had higher blood pressure than controls, while no such difference was found in the group with high mtDNA function.

**Conclusion:**

Increased mtDNA copy number and decreased D-loop and *MT-TF* methylation were associated with particle-containing welding fumes exposure, indicating exposure-related oxidative stress. The modification of mtDNA function on exposure-associated increase in blood pressure may represent a mitochondria-environment interaction.

**Electronic supplementary material:**

The online version of this article (doi:10.1186/s12940-017-0234-4) contains supplementary material, which is available to authorized users.

## Background

In work environments, the welding process is an important emission source of fine and ultrafine particles with a mass median diameter of 200–300 nm [1]. The reaction between vaporized metals and air during welding produces different types of metal oxides, including iron (Fe), manganese (Mg), chromium (Cr), and nickel (Ni) that result in the complex chemical properties of welding fume [2]. Studies have found associations between exposure to welding fumes and chronic obstructive pulmonary disease [3, 4], lung cancer [5] and various cardiovascular diseases [6, 7]. In the current project, we have reported higher blood pressure in the welders than in the controls, and that years of working as a welder were associated with increased blood pressure [8]. Growing evidence suggested that oxidative stress could be an intermediate step linking welding fumes exposure and disease [9–11]. Oxidative stress induced by particles with metal components (e.g. Cr, Ni, Fe) has been consistently shown to alter the methylation level of nuclear DNA and cause DNA damage [12–15].

Mitochondria, located in all types of cells except in red blood cells, are unique organelles with the primary biological function of generating energy [16]. They carry their own extranuclear, closed circular double-strand DNA: mitochondrial DNA (mtDNA). MtDNA is more susceptible to oxidative stress than nuclear DNA due to a lack of histones for protection, less adequate DNA repair capacity, and close proximity to the electron transport chain [17]. The copy numbers of mtDNA vary in each mitochondrion, as well as in different cells, different tissues and individuals. Alteration of mtDNA copy number has been observed as a response to oxidative stress in vitro and in vivo [18–20]. Moreover, integrity of the mitochondrial genome can affect mitochondrial function [21]. MtDNA encodes 13 respiratory chain polypeptides, 22 transfer RNAs (tRNA) and 2 ribosomal RNAs (rRNA) [22], and has a noncoding control region called the displacement loop (D-loop). The presence of mtDNA methylation has been debated for decades. An in vivo study of mtDNA – protein interaction observed methylation in the mitochondrial genome [23]. Then, Shock et al. demonstrated an enrichment of 5-methylcytosine and 5-hydroxymethylcytosine together with the presence of DNA methyltransferases 1 inside mitochondria [24]. More recently, Bellizzi et al. confirmed that mtDNA is indeed methylated, particularly in the D-loop region [25]. The D-loop contains three promoters required for transcription initiation and nearly the entire mitochondrial genome transcribes from this region [26]. Another gene of interest for mtDNA epigenetics is the transfer RNA phenylalanine (*MT-TF*) gene that encodes a tRNA involved in intra-mitochondrial translation and essential for protein synthesis [22]. Mitochondria play a crucial role for regulation of energy generation, and redox signaling of cells in the cardiovascular system [27], and thus, indicate that mitochondrial function is important for cardiovascular health. Still, there is limited knowledge how environmental exposures modify mtDNA epigenetics. It is also reasonable to infer that the mtDNA function might modify the relationship between exposure to particles and adverse cardiovascular effects.

In the present study, we aimed to elucidate the effects of occupational exposure to welding fumes on mtDNA copy number, methylation in the D-loop region and *MT-TF* gene. Furthermore, we also attempted to explore if these mitochondrial markers can modify the association between welding fumes and cardiovascular response, measured as blood pressure.

## Methods

### Study participants

Details of study participants’ recruitment have been reported previously [8]. In short, we investigated 101 welders and 127 controls (male and currently non-smoking) from southern Sweden. All participants had been non-smoking for more than one year. The welders used the same gas metal arc welding method; therefore they were exposed to relatively homogenous compositions of welding fumes. Fifty-five % of the welders reported that they used local exhaust ventilation, 56% reported that they use only welding shields as personal protection and 44% reported that they used powered air purifying respirators. All the welders also used protective clothing. The non-exposed controls were mainly workers in storage rooms, uploading and offloading products, and thus, their physical workload was comparable to the welders in our study.

All participants went through a structured face-to-face interview regarding potential particle exposure (e.g. particle from wood burning at home, traffic intensity outside their house windows), disease history (personal and family), and daily life (e.g. smoking history, daily diet, and activity and/or training). The answers were categorized into several groups. After the interviews, the participants went through blood pressure measurements and blood sampling. The study was approved by the Regional Ethical Committee of Lund University. All study participants gave fully informed and signed consent for their participation.

### Exposure assessment

We used respirable dust as the indicator of exposure to particles from welding fumes [28]. Personal sampling of respirable dust was performed in the workers’ breathing zone for 70 welders (among them, 17 welders were not participants in the medical part of the study) at the work places of 10 welding companies in the manufacturing industry by an occupational hygienist by use of pre-weighed 37 mm mixed cellulose ester filters (0.8 μm pore size) fitted in conductive cassettes attached to cyclones (BGI4L, BGI Inc., USA; 50% cutoff at an aerodynamic equivalent particle diameter of 4 μm). The air flow was set at 2.2 L/min, and was regularly checked with a primary calibrator (TSI Model 4100 Series, TSI Inc., Shoreview, MN, USA) before, during, and after the sampling. The personal sampling was also performed for 19 controls from two control companies. Real-time measurements of background particle mass concentrations were conducted in other four control companies with direct reading instrument (Sidepak Model AM510, TSI Inc., MN USA).

Most of the personal sampling was performed during full-shift work with an average 6.9 h sampling time (range 2.4-8.6 h, only 5 out of 70 welders had sampling time shorter than 4 h). The filters were analyzed gravimetrically for respirable dust therefore the concentrations were the accumulation of the full-shift work. If the welders used powered air purifying respirators, the air outside the respirators was sampled, and the measured concentrations were reduced by a correction factor of 3 to get a better estimation of the exposure inside the respirator [29]. For the 48 welders with no exposure measurements, the exposure to respirable dust was estimated from the personal exposure data of welders (*n* = 70) with similar tasks.

### Blood pressure and blood sampling

Each participant was asked to be seated during the 15 min structured interview. Blood pressure was then measured once by the skilled occupational health nurse using a mercury sphygmomanometer, with an adjustable cuff corresponding to different arm circumference in supine position. Peripheral blood was obtained afterwards, transported to the laboratory on dry ice, and stored at −20 °C until extraction of DNA.

### Analysis of relative mitochondrial DNA copy number (RmtDNAcn)

DNA was isolated from whole peripheral blood by Qiagen DNA Blood Midi kit (Qiagen, Heidelberg, Germany). An assay based on real-time quantitative polymerase chain reaction (PCR) and SYBR® Green technology was adopted to determine mtDNA copy number relative to the single copy hemoglobin beta (*HBB*) gene using two independent PCRs. Master mixes for mtDNA copy number and *HBB* were prepared with KAPA SYBR FAST qPCR Kit Master Mix (2X) ABI Prism (Kapa Biosystems, Woburn, MA, USA) and corresponding primers (0.20 μM for each primer). Primers for mtDNA were: forward 5′-CAC CCA AGA ACA GGG TTT GT-3′ and reverse 5′-TGG CCA TGG GTA TGT TGT TA-3′; and primers for the *HBB* gene were: forward 5′-TGT GCT GGC CCA TCA CTT TG-3′ and reverse 5′-ACC AGC CAC CAC TTT CTG ATA GG-3′, as previously described [30]. PCR was performed on a real-time PCR machine (7900HT, Applied Biosystems, Foster City, CA, USA). Each reaction (end volume 10 μL) consisted of 2.5 μL of DNA (4 ng/μL) and 7.5 μL master mix. The thermal cycle profile was 95 °C for 3 min, followed by 95 °C for 3 s and 60 °C for 20 s for 25 cycles (mtDNA) or 35 cycles (*HBB*). A standard curve and a blank were included in each run. For the standard curve, one reference DNA sample (a pool of 20 samples randomly picked) was diluted serially by twofold per dilution to produce 5 concentrations of 1 – 16 ng/μL. A control sample was also included in each run to monitor the variance between runs. All samples and standard curve points were run in triplicates. R^2^ for each standard curve was >0.99. Standard deviations of triplicates <0.1 were accepted for the C_t_ values. SDS 2.4.1 software (Life Technologies) calculated the relative quantity of mtDNA and *HBB* for each sample, based on the standard curve. The relative mtDNA copy number (RmtDNAcn) was the quotient of the relative quantity of mtDNA and HBB, thus, it is an arbitrary value. The coefficient of variation (CV) of the control sample was 14% based on 3 runs. The raw data of individual RmtDNAcn is provided as Additional file 1.

### Analysis of mitochondrial DNA methylation

Bisulfite modification was performed on 500 ng of peripheral blood DNA with EZ-96 DNA Methylation-Gold kit (Catalogue number D5008; Zymoresearch, Irvine, CA) according to the manufacturer’s instructions. We used 0.6 μl bisulfite-treated DNA in a 15 μl PCR reaction using the Pyromark PCR kit (Qiagen). The customer designed PCR primers and sequencing primers for the assay of D-loop and *MT-TF* methylation were described by Byun et al., and reverse PCR primers were biotinylated [31]. PCR was performed using PyroMark PCR reagents (Qiagen, catalog nr 972807). Detailed PCR conditions and primer sequences for mtDNA methylation assays are listed in Additional file 2: Table S1. The PCR products of 24 randomly picked samples were tested by gel and no non-specific binding was noticed. The PCR products were purified using Streptavidin Sepharose High Performance beads (Amersham Biosciences, Uppsala, Sweden). The Sepharose beads with the immobilized PCR products were purified, washed, and denatured with 0.2 M NaOH and washed again using a vacuum prep tool (Pyrosequencing Inc., Westborough, MA, USA). We performed pyrosequencing using the PSQ HS96 Pyrosequencing System (Qiagen). Negative PCR control (without DNA template) was included in four test plates and no signal of DNA methylation was measured in any of the negative controls. The degree of DNA methylation was expressed as the percentage of methylated cytosines over the sum of methylated and unmethylated cytosines (% DNA methylation). Three CpG sites from the D-loop region and one CpG site from the *MT-TF* gene were measured. We took an average percentage of methylation of D-loop for the analyses since the three sites were highly correlated (*r*_*S*_ range 0.71-0.78). The raw methylation data of four CpG sites is provided as Additional file 1. We repeated eight samples and found the coefficient of variation as 12% for D-loop and 22% for *MT-TF*.

### Statistical analysis

Basic characteristics like age, BMI, and blood pressure, as well as mtDNA biomarkers were symmetrically distributed for welders and controls and compared by t-test, while the exposure to respirable dust was skewed between the two groups and compared by Mann–Whitney U tests. Tobacco use (proportions of previous smokers, use of the smokeless tobacco “snus”, a moist crushed tobacco placed under the upper lip), and mtDNA function group (for details of calculation see below) were compared by Fisher’s exact tests. Pearson correlation coefficients were used to evaluate the correlations between RmtDNAcn, D-loop, and *MT-TF* methylation.

#### Exposure, copy number and methylation of mtDNA

General linear models were used to analyze the effects of occupational group (welders vs. controls), or exposure level (continuous variables: personal exposure to respirable dust, and years of working as a welder) on RmtDNAcn, D-loop and *MT-TF* methylation. Potential confounders (age, BMI, variables regarding smoking history, family history of disease, potential particle exposure, daily diet, alcohol consumption and activity and/or training) were chosen based on published studies and general knowledge, and tested one by one in the models. Only the confounders which changed the β-estimate of exposure indexes by more than 10% remained in further analyses. Therefore, age, BMI, previous smoking years, smokeless tobacco “snus” status and current residence were included in the adjusted models.

#### Calculation of mtDNA function

To investigate the modification of mtDNA function, we considered markers representing both the copy number and the methylation of mtDNA. We calculated the relative number of unmethylated mtDNA in the D-loop region and *MT-TF* gene. This was performed by calculating unmethylated D-loop and unmethylated *MT-TF* separately by subtracting the percentage of methylation of each region from 1. Then, we multiplied the products of RmtDNAcn and unmethylated D-loop (relative number of unmethylated D-loop), or RmtDNAcn and unmethylated *MT-TF* (relative number of unmethylated *MT-TF*), respectively, and used the products as the markers of mtDNA function. The formulas were:

$$ \begin{array}{l}\mathrm{Relative}\ \mathrm{number}\ \mathrm{of}\ \mathrm{unmethylated}\ \mathrm{D}\hbox{-} \mathrm{loop} = \left(1\ \hbox{-}\ \mathrm{methylated}\ \mathrm{D}\hbox{-} \mathrm{loop}\ \%\right) \times \mathrm{relative}\ \mathrm{mtDNA}\ \\ {}\mathrm{copy}\ \mathrm{number}.\end{array} $$

$$ \begin{array}{l}\mathrm{Relative}\ \mathrm{number}\ \mathrm{of}\ \mathrm{unmethylated}\kern0.5em  MT\mathit{\hbox{-}} TF = \left(1\ \hbox{-}\ \mathrm{methylated}\kern0.5em  MT\mathit{\hbox{-}} TF\%\right) \times \mathrm{relative}\ \mathrm{mtDNA}\ \\ {}\mathrm{copy}\ \mathrm{number}.\end{array} $$

Therefore, higher mtDNA function represented more copies of active (unmethylated) mtDNA, showing as greater relative number of unmethylated D-loop or *MT-TF*.

#### Modification of mtDNA function

First, interaction terms of mtDNA function markers and occupational group/exposure level were introduced into the general linear models to see if there was a modifying effect on mtDNA function. Then, data was stratified in two subgroups by the median value of mtDNA function (low and high mtDNA function groups). Age, BMI, family history of cardiovascular disease, smoking history, and current residence were selected as adjustments based on the same criteria of inclusion.

The residuals from each linear regression model were examined and all showed symmetric distribution. All statistical analyses were completed by SPSS (Version 22.0; IBM SPSS Statistics for Windows, NY, USA).

## Results

### Characteristics and biomarkers of study participants

Age (*p* = 0.94) and BMI (*p* = 0.70) were similar in the welders and the controls, as were the proportions of previous smoking (*p* = 0.21) and the average years of previous smoking (*p* = 0.52). The smokeless tobacco “snus” status was not different between two occupational groups (*p* = 0.15, 28% in the welders and 19% in the controls). The average concentration of respirable dust was 1.1 mg/m^3^ in the welders, while no participant in the control group was exposed to respirable dust above 0.1 mg/m^3^. RmtDNAcn was higher in the welders than in the controls (*p* = 0.0049), and D-loop and *MT-TF* methylation were lower in the welders than the controls (*p* = 0.0012 for D-loop, *p* = 0.0015 for *MT-TF*; Table 1). When stratifying study participants into low/high mtDNA function groups based on the median value of all participants, we found more welders in the high mtDNA function group, while more controls in the low mtDNA function group (*p* = 0.026 for D-loop, *p* < 0.001 for *MT-TF*; Table 1).

**Table 1:** Basic characteristics and mitochondrial DNA biomarkers in welders (*n* = 101) and controls (*n* = 127)

	Welder	Control	*p* ^g^
Age^a^	41 (23 – 60)	43 (23 – 56)	0.94
BMI (kg/m^2^)^a^	28 (22 – 34)	27 (22 – 34)	0.70
Respirable dust (mg/m^3^)^a,b^	1.1 (0.2 – 8.4)	0.1 (0.0 – 0.1)	<0.001^h^
Working years as welder (year)^a^	15 (1 – 38)	---	--
Systolic blood pressure (mm Hg)^a^	130 (115 – 155)	125 (105 – 145)	<0.001
Diastolic blood pressure (mm Hg)^a^	75 (60 – 85)	70 (60 – 85)	<0.001
Previous smoking (yes/no (%))	43/58 (43%)	43/83 (34%)	0.21^i^
Previous smoking years (year)^a^	10 (3 – 32)	14 (2 – 31)	0.52
Smokeless tobacco “snus” (yes/no (%))	28/73 (28%)	24/103 (19%)	0.15^i^
Relative mtDNA copy number^a^	1.13 (0.84 – 1.52)	1.00 (0.74 – 1.50)	0.0049
D-loop methylation^a, c^	13.4 (10.5 –20.8)	15.6 (10.9 – 21.1)	0.0012
*MT-TF* methylation^a, c^	3.4 (0 – 9.5)	4.5 (0 – 12.1)	0.0015
mtDNA function group (high/low (%)) (D-loop)^d, f^	57/37 (60%)	51/66 (44%)	0.026^i^
mtDNA function group (high/low (%)) (*MT-TF*)^e, f^	63/37 (63%)	47/73 (39%)	<0.001^i^

^a^Presented as median (5 – 95 percentile)

^b^Median of respirable dust in welder group was based on 70 welders with measured respirable dust

^c^Median of D-loop methylation was based on 211 participants (94 welders and 117 controls); median of *MT-TF* methylation was based on 220 participants (100 welders and 120 controls)

^d^Derived from unmethylated D-loop and relative mtDNA copy number: Relative number of unmethylated D-loop = (100% - % methylated D-loop) × relative mtDNA copy number

^e^Derived from unmethylated *MT-TF* and relative mtDNA copy number: Relative number of unmethylated *MT-TF* = (100% - % methylated *MT-TF*) × relative mtDNA copy number

^f^Stratified into low and high groups based on the median. Here, we present the count (%) of participants in each function group

^g^ *p* value obtained from t test unless marked otherwise

^h^ *p* values obtained from Mann–Whitney U test

^i^ *p* values obtained from Fisher’s exact test

**Fig. 1:**
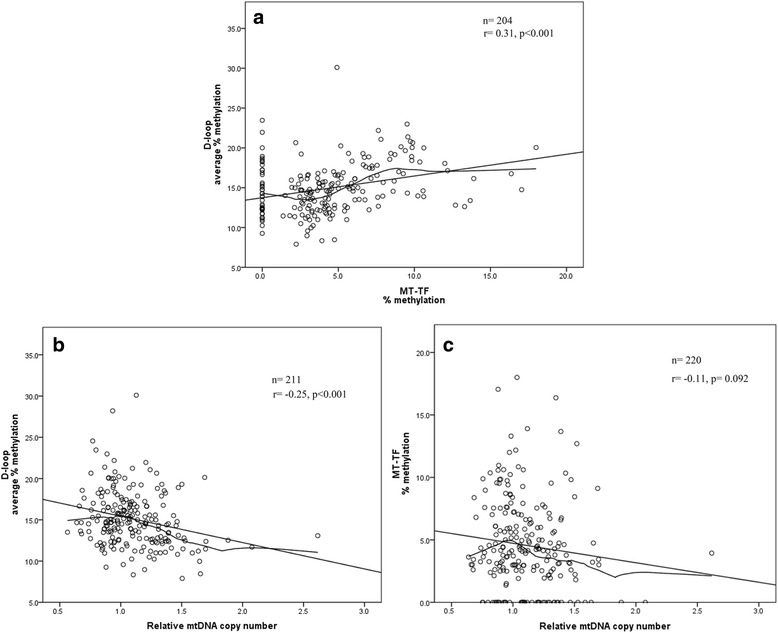
Correlations between methylation and relative mtDNA copy number. Scatterplots with linear and loess fit lines showing the correlations between mitochondrial gene methylation and relative mtDNA copy number: **a** D-loop methylation and *MT-TF* methylation; **b** D-loop methylation and relative mtDNA copy number; **c** *MT-TF* methylation and relative mtDNA copy number

D-loop methylation was positively correlated with *MT-TF* methylation (*r* = 0.31, *p* < 0.001; Fig. 1a), and inversely correlated with RmtDNAcn, (*r* = −0.25, *p* < 0.001; Fig. 1b). No correlation between *MT-TF* methylation and RmtDNAcn was found (*r* = −0.11, *p* = 0.092; Fig. 1c).

### Associations between exposure to welding fumes and mtDNA copy number and methylation

**Fig. 2:**
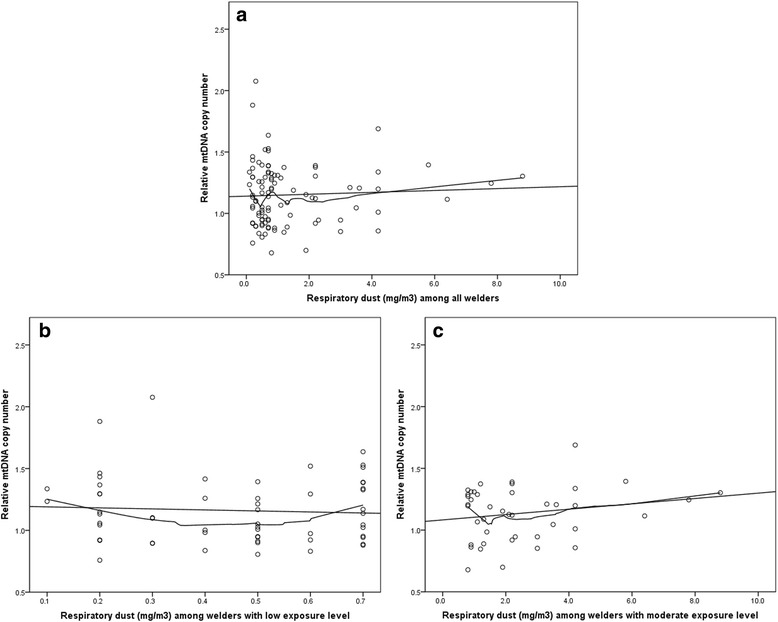
Associations between relative mtDNA copy number and personal respirable dust. Scatterplots with linear and loess fit lines showing the associations between respirable dust and relative mtDNA copy number. Associations are (**a**) among all welders, (**b**) among welders with exposure level lower than 0.7 mg/m^3^ (median concentration = 0.5 mg/m^3^) and (**c**) among welders with exposure level higher than 0.7 mg/m^3^ (median concentration = 2.0 mg/m^3^). The cut-off of exposure level was based on median value of respirable dust from 101 welders (including measured and estimated exposures)

The differences of RmtDNAcn and mtDNA methylation status between welders and controls remained significant after adjustment for age, BMI, previous smoking years, smokeless tobacco “snus” status, and current residence (Table 2, unadjusted model see Additional file 3: Table S2). In order to elucidate dose-effect relationships of welding fume particles on RmtDNAcn and mtDNA methylation, analyses were performed in welders only. Figure 2 shows the associations between RmtDNAcn and personal respirable dust, indicating a slightly different pattern of dose-effect relationship among welders with different exposure levels: RmtDNAcn was weakly positively associated with respirable dust among welders with personal exposure level above 0.7 mg/m^3^ (β = 0.037, *p* = 0.054; Table 2), however, no clear association was found among welders with low exposure level (below 0.7 mg/m^3^). No associations were observed for personal respirable dust concentrations and D-loop methylation or *MT-TF* methylation (figures not shown). No association between RmtDNAcn, mtDNA methylation and years of working as a welder was found (Table 2).

**Table 2:** Associations between exposure and relative mtDNA copy number, D-loop and *MT-TF* methylation in adjusted models^a^

	mtDNA copy number			D-loop methylation			*MT-TF* methylation		
	N	Beta (95% CI)	*p*	N	Beta (95% CI)	*p*	N	Beta (95% CI)	*p*
Occupational group									
Welders vs. controls^b^	228	0.11 (0.037, 0.18)	0.0032	211	−1.4 (−2.3, −0.5)	0.0021	220	−1.5 (−2.5, −0.48)	0.0038
Exposure level									
Respirable dust^c^	101	0.017 (−0.018, 0.052)	0.33	92	0.012 (−0.47, 0.50)	0.96	97	0.097 (−0.32, 0.51)	0.64
Respirable dust (<=0.7 mg/m^3^)^c, d^	57	−0.031 (−0.47, 0.41)	0.89	54	−0.42 (−4.76, 3.9)	0.85	56	2.9 (−2.2, 8.1)	0.26
Respirable dust (>0.7 mg/m^3^)^c, d^	41	0.037 (−0.00075, 0.075)	0.054	38	−0.043 (−0.85, 0.76)	0.92	41	0.17 (−0.29, 0.63)	0.46
Working years^e^	100	−0.0027 (−0.010, 0.0049)	0.49	93	0.061 (−0.053, 0.16)	0.29	99	−0.013 (−0.10, 0.076)	0.77

^a^The adjusted model included age, BMI, previous smoking years, smokeless tobacco “snus” status and current residence as adjustments

^b^Effect estimates presented are β-values for occupation (welders compared with control) derived from general linear models

^c^Effect estimates presented are β-values for personal respirable dust (only welders included) derived from general linear models

^d^The cut-off was based on median value of welders with measured and estimated respirable dust

^e^Effect estimates presented are β-values for years working as welder (only welders included) derived from general linear models

### Interaction of mtDNA function with exposure on blood pressure

There was some indication of interaction between the occupational groups and the mtDNA function marker (relative number of unmethylated D-loop) on blood pressure (for systolic blood pressure: interaction *p* = 0.15, for diastolic blood pressure, interaction *p* = 0.20). Similarly, interactions were suggested between the occupational groups and relative number of unmethylated *MT-TF* (*p* = 0.073 for systolic blood pressure, *p* = 0.25 for diastolic blood pressure). The suggested interactions may indicate a modifying effect of mtDNA function on the associations between occupational groups and blood pressure. Thus, we stratified the study participants into low and high mtDNA function groups by the median of relative number of unmethylated D-loop. The high mtDNA function group represented more copies of active (unmethylated) D-loop than the low function group. After stratifying the data, we found higher systolic blood pressure in the welders than the controls (β = 11.3, *p* < 0.001) in the group with low mtDNA function, but not in the high group (β = 3.3, *p* = 0.15, Table 3). Similarly, after stratifying the data by the relative number of unmethylated *MT-TF*, higher systolic blood pressure in the welders than in the controls (β = 12.3, *p* < 0.001, Table 3) was only found in the group with low mtDNA function. However, no modifying effects of mtDNA function on the association between blood pressure and years of working as a welder, or between blood pressure and respirable dust, were found (data not shown).

**Table 3:** Associations between occupational groups and blood pressure in different mtDNA function groups^a^

	mtDNA function group (D-loop)^b^						mtDNA function group *(MT-TF)* ^c^					
	Low			High			Low			High		
Outcome	N	Beta (95% CI)	*p*	N	Beta (95% CI)	*p*	N	Beta (95% CI)	*p*	N	Beta (95% CI)	*p*
Systolic blood pressure	100	11.32 (6.61, 15.91)	<0.001	106	3.34 (−1.18, 7.85)	0.15	107	12.31 (7.93, 16.69)	<0.001	108	1.11 (−3.51, 5.73)	0.63
Diastolic blood pressure	100	3.53 (0.18, 6.88)	0.039	106	3.64 (0.28, 7.00)	0.034	107	3.00 (−0.38, 6.38)	0.081	108	2.39 (−0.96, 5.73)	0.16

^a^The model included age, BMI, family history of cardiovascular disease, smoking history, and current residence as adjustments

^b^Derived from unmethylated D-loop and relative mtDNA copy number. Two groups stratified by median

^c^Derived from unmethylated *MT-TF* and relative mtDNA copy number. Two groups stratified by median

## Discussion

In this study, we hypothesized that occupational exposure to particles could be associated with alteration in mtDNA function, and mtDNA function may interact with exposure on adverse cardiovascular effects, measured as blood pressure. We found that the occupation as welder was associated with changes in mtDNA, both measured as copy number and the methylation status of key regions of the mtDNA, indicating an effect of welding fumes on the mitochondrial function. There was, however, no strong dose-effect relationship between personal respirable dust or working years as a welder and biomarkers of mtDNA function, and these findings need therefore to be interpreted cautiously. The changes in mtDNA may affect mitochondrial biogenesis and cellular function, and in turn contribute to higher risk of cardiovascular diseases through interaction with particle exposures.

There is a widespread interest in exploring the response of mtDNA to environmental exposures. Recent studies have shown a linkage between particle exposure and alteration in mtDNA in peripheral blood, but the results are inconsistent [30–34]. Hou et al. found particle-related increased mtDNA copy number in 63 steel workers at time-weighted PM_1_ concentration around 9 μg/m^3^ [30]. However, the same research group reported that exposure to elemental carbon at around 15 μg/m^3^ and ambient PM_10_ at around 120 μg/m^3^ during work hours, was related to decreased mtDNA copy number. No association was found between personal exposure to PM_2.5_ and mtDNA copy number [32]. One cross-sectional study involving 20 steel workers and 20 controls reported a positive association between metal-rich particle exposure and mtDNA methylation in *MT-TF* and *MT-RNR1* region encoding 12 s rRNA but they did not find any association between particle exposure and D-loop methylation [31]. Yet, a recent paper involving 48 healthy male workers reported a negative association between PM_2.5_ and D-loop methylation, but not in the *MT-TF* and *MT-RNR1* region [34].

In the present study, we observed higher RmtDNAcn together with lower methylation levels in the mtDNA D-loop region in peripheral blood in the welders than the controls, and a weak positive dose-effect relationship between personal respirable dust and RmtDNAcn among the welders with moderate exposure level. The inconclusive results between the studies might be due to the different size and compositions of particles, different exposure concentrations in each study, as well as small study size resulting in low power. The mtDNA synthesis can be stimulated by mild oxidative stress, resulting in an increase of mtDNA copy number to supply energy for cell survive, while immoderate oxidative stress may lead to decrease in mtDNA synthesis because of defect mitochondria and result in apoptosis and cell death [30, 35]. Since the median particle concentration in our study (1.1 mg/m^3^) is not very high (the occupational exposure limit is set as 5 mg/m^3^ by the Swedish Work Environment Authority [28]), we concluded that the increase in RmtDNAcn could be a feedback response to compensate defect mitochondria with an impaired respiratory chain, or mutated mtDNA caused by oxidative damage from welding fume [18, 35]. The methylation level change in the D-loop could be another molecular event related to particle induced oxidative stress, which can damage methylation of nucleotides [36]. Classic epigenetic theory suggests that hypermethylation in the promoter region are generally transcriptionally silent, while demethylated DNA is usually associated with active gene expression [37, 38]. Based on the finding that demethylated mtDNA D-loop was associated with increased ND2 expression (a subunit of NADH, a rate-limiting enzyme of oxidative phosphorylation), which can regulate the generation of ATP [39], our findings of demethylation of D-loop in the welders could be interpreted as mtDNA response to cope with the increased oxidative stress by increasing energy production related gene expression to maintain normal cellular function. Thus, both the increase in RmtDNAcn and the decrease in methylation level in welders could be the compensatory mechanisms of mtDNA responses to particle exposure. Recently, Janssen et al. studied mtDNA methylation and mtDNA content in placental tissues in the context of the early life exposure to environmental airborne particles, and they observed opposite effects of particle exposure on mtDNA: i.e. an inverse association between PM_2.5_ and mtDNA content, as well as a positive association with mtDNA methylation in D-loop and *MT-RNR1* [40]. The contradictory finding between our study and theirs could be due to the duration of exposure that the mothers in their study were exposed to ambient PM_2.5_ during the entire pregnancy, while the exposure to respirable dust was only measured once in our study. Another possibility is that the different origin of DNA matters. Given the fact that the placenta is an organ that requires a constant and abundant source of energy, mtDNA in placenta may response more readily to particle exposure compared to mtDNA in blood, and therefore, produce more by-product reactive oxygen species that in turn more rapidly causes mtDNA damage.

Despite the noticeable findings of occupational group, the effect of exposure levels on mtDNA was not statistically certain. One explanation about the small effect estimates of respirable dust could be that other unmeasured exposures, like ozone production in gas phase during the welding process [2], might have been responsible for the increase in RmtDNAcn. We also failed to identify dose–response associations among welders with low exposure, which could suggest a threshold of particle exposure for mtDNA effects. However this hypothesis remains unconfirmed as we only carried out respirable dust sampling once, and it is dangerous to assume that one measurement can represent the long-term exposure dose.

The association between particle exposure and higher blood pressure has been suggested to be caused by the generation of reactive oxygen species, which interrupt the redox state, cause systemic inflammation and induce endothelial dysfunction and vascular injury in hypertension development [41, 42]. Given the fact that mitochondria are important to cellular function, mtDNA function may play a critical role in the etiology of particle-related hypertension. Our finding of possible modification of mtDNA function on exposure and blood pressure indicated that individuals carrying lower copies of active mtDNA may be a susceptible group, possibly because of insufficient energy generation and antioxidant capacity. The vicious cycle of decreased mitochondrial antioxidant capacity and continuous oxidative stress could induce endothelial dysfunction and vascular smooth muscle cell hypertrophy [43], which are the main characteristic alterations of vascular wall in hypertension [44]. We are aware that our grouping of low and high mtDNA function was rather crude; further follow-up studies are required to determine the effects of changes in mtDNA on mitochondrial function.

The primary limitation of the study was the cross-sectional study design, which precludes conclusions with regard to causality. However, the information from the structured interview allowed proper control of possible confounders in the statistical analysis to avoid biased associations. The single measurement of RmtDNAcn, mtDNA methylation did not permit us to determine intrapersonal variation over time. In the study, we only measured the blood pressure once for each subject, due to the practical circumstance that workers were busy and could not spare more time for medical examination. However, blood pressure measurement in this study served as an indicator of adverse cardiovascular effects, rather than a clinical diagnosis of hypertension. We cannot confirm the direct linkage between particle exposure, methylation and its functional significance on RNA expression since RNA was not available. This would be an interesting topic for further research. The relatively higher CV for *MT-TF* methylation is probably due to the low methylation of this CpG, which may be influenced by noise of the assay. Therefore, the changes in *MT-TF* methylation needs to be validated in further studies.

We also recognize that mtDNA methylation in this paper is likely overestimated. One recently published paper revealed that measurement of mtDNA methylation level is affected by its form: circular mtDNA appears to have higher methylation level than linear mtDNA, due to incomplete bisulfite conversion when mtDNA is circular [45]. In our study, we did not break the circular DNA into linear, and obtained an average D-loop methylation around 13 - 16%, which is similar to Liu et al. (11.65% ± 0.95%) [45] when circular mtDNA was not broken. However, we do not consider it is likely that the difference in methylation between circular and linear mtDNA would influence our conclusions. Both controls and welders were sampled in the same way and analyzed, in a randomized set-up, by the same mtDNA method; therefore the systematic overestimation apply to all study participants in the study, and should not change the relative comparison between exposed group and control group.

## Conclusion

In summary, we observed a potential effect of occupational exposure to particles on mtDNA in welders, although the occupational exposure level to particles was only low to moderate. The modification of mtDNA function on the welding-associated increase in blood pressure, may represent a mitochondria-environment interaction, and may indicate that mtDNA plays a critical role in the etiology of particle-related cardiovascular disease. Studies further disentangling these associations may be an important step in understanding the mechanisms behind particle-induced cardiovascular disease.

## Additional files

Additional file 1: Raw data of mtDNA copy number and methylation in D-loop and *MT-TF* in Excel. (XLSX 21 kb)
Additional file 2: Table S1. Detailed PCR conditions and primer sequences for mtDNA methylation assays. (DOCX 17 kb)
Additional file 3: Table S2. The associations between occupational group/exposure level and relative mtDNA copy number, D-loop and MT-TF methylation in unadjusted models. (DOCX 18 kb)

## Abbreviations

Cr: Chromium
D-loop: Displacement loop
Fe: Iron
*HBB*: Hemoglobin beta
Mg: Manganese
mtDNA: Mitochondrial DNA
*MT-TF*: Transfer RNA phenylalanine
Ni: Nickel
PCR: Polymerase chain reaction
RmtDNAcn: Relative mitochondrial DNA copy number
