# Supplementary material for: Occupational exposure to particles and mitochondrial DNA - relevance for blood pressure
Source: Environ Health. 2017 Mar 9;16:22. doi: 10.1186/s12940-017-0234-4 (PMC5343309; doi:10.1186/s12940-017-0234-4)
Supplement: Additional file 2: Table S1. — Detailed PCR conditions and primer sequences for mtDNA methylation assays. (DOCX 17 kb) [file 12940_2017_234_MOESM2_ESM.docx]

Table S1: Detailed PCR conditions, primer sequences and analyzing sequence for Bisulfite-Pyrosequencing

| Primer | Sequence | Target CpGs | PCR conditions |
| --- | --- | --- | --- |
| MT-TF (F) | 5’-TAAAGTAATATATTGAAAATGTTTAGA-3’ | 1 CpG | 95℃ for 15 min and 45 cycles of denaturation at 94℃ for 30 sec; annealing at 53℃ for 30 sec and extension at 72℃ for 30 sec. |
| MT-TF (R) (bio) | 5’-TACTTAATACTTATCCCTTTTAATC-3’ |  |  |
| MT-TF  Sequencing primer | 5’-TATTGAAAATGTTTA-3’ |  |  |
| D-loop (F) | 5’-TGTGTAGATATTTAATTGTTATTA-3’ | 3 CpGs |  |
| D-loop (R) (bio) | 5’-CAAATCTATCACCCTATTAACCAC-3’ |  |  |
| D-loop  Sequencing primer | 5’-TAATTAATTAATATATTT-3’ |  |  |
|  | | | |
| Sequence to analyze (for pyrosequencing) | | | |
| MT-TF | GACGGGTTTATATTATTTTATAAATAAAT | | |
| D-loop | TAGTAAGTATGTTCGTTTGTAATATTGAACGTAGGTGCGATAAAT | | |
