# Supplementary material for: Occupational exposure to particles and mitochondrial DNA - relevance for blood pressure
Source: Environ Health. 2017 Mar 9;16:22. doi: 10.1186/s12940-017-0234-4 (PMC5343309; doi:10.1186/s12940-017-0234-4)
Supplement: Additional file 3: Table S2. — The associations between occupational group/exposure level and relative mtDNA copy number, D-loop and MT-TF methylation in unadjusted models. (DOCX 18 kb) [file 12940_2017_234_MOESM3_ESM.docx]

**Table S2. The associations between occupational group/exposure level and relative mtDNA copy number, D-loop and *MT-TF* methylation in unadjusted model.**

|  | mtDNA copy number | | D-loop methylation | | *MT-TF* methylation | |
| --- | --- | --- | --- | --- | --- | --- |
|  | Beta  (95% CI) | P | Beta  (95% CI) | P | Beta (95% CI) | P |
| **Occupational group** | | | | | | |
| Welders vs. controls ^a^ | 0.098  (0.030, 0.17) | 0.0050 | -1.5  (-2.4, -0.6) | 0.0012 | -1.6  (-2.5, -0.60) | 0.0015 |
| **Exposure level** |  |  |  |  |  |  |
| Respirable dust ^b^ | 0.0078  (-0.023, 0.038) | 0.62 | 0.091  (-0.35, 0.52) | 0.70 | -0.018  (-0.38, 0.35) | 0.92 |
| Respirable dust  (<=0.7 mg/m^3^) ^b, c^ | -0.083  (-0.44, 0.30) | 0.65 | -0.62  (-4.8, 3.5) | 0.77 | 1.8  (-2.6, 6.2) | 0.42 |
| Respirable dust  (>0.7 mg/m^3^) ^b, c^ | 0.022  (-0.013, 0.056) | 0.21 | -0.0051  (-0.68, 0.67) | 0.99 | -0.042  (-0.44, 0.36) | 0.83 |
| Working years ^d^ | -0.00034  (-0.0051, 0.0044) | 0.89 | 0.052  (-0.022, 0.12) | 0.15 | 0.0067  (-0.050, -0.064) | 0.82 |

^a^ Effect estimates presented are β-values for occupation (welders compared with control) derived from general linear model.

^b^ Effect estimates presented are β-values for personal respirable dust (only welders included) derived from general linear models.

^c^ The cut-off was based on median value of welders with measured and estimated respirable dust.

^d^ Effect estimates presented are β-values for years working as welder (only welders included) derived from general linear models.
